# APASL and AASLD Consensus Guidelines on Imaging Diagnosis of Hepatocellular Carcinoma: A Review

**DOI:** 10.4061/2011/519783

**Published:** 2011-04-19

**Authors:** Cher Heng Tan, Su-Chong Albert Low, Choon Hua Thng

**Affiliations:** ^1^Department of Diagnostic Radiology, Tan Tock Seng Hospital, 11 Jalan Tan Tock Seng, Singapore 308433; ^2^Department of Diagnostic Radiology, Singapore General Hospital, Outram Road, Singapore 169608; ^3^Department of Oncologic Imaging, National Cancer Centre, 11 Hospital Drive, Singapore 169610

## Abstract

Consensus guidelines for radiological diagnosis of hepatocellular carcinoma (HCC) have been drafted by several large international working groups. This article reviews the similarities and differences between the most recent guidelines proposed by the American Association for Study of Liver Diseases and the Asian Pacific Association for the Study of the Liver. Current evidence for the various imaging modalities for diagnosis of HCC and their relevance to the consensus guidelines are reviewed.

## 1. Introduction

Consensus guidelines have been drafted by several large international working groups on different occasions in an attempt to standardise the surveillance, diagnosis, and management of HCC. Of the major working groups, the European Association for the Study of the Liver was the first to establish consensus guidelines on the clinical management of HCC following the Barcelona European Association for the Study of the Liver (EASL) Conference in 2000 [1]. The American Association for Study of Liver Diseases (AASLD) adapted these recommendations to issue a set of consensus recommendations in 2005 [2]. This was more recently updated in 2010 [3]. The Asian Pacific Association for the Study of the Liver (APASL) itself also developed a set of consensus recommendations in December 2008 [4]. 

The rationale for a set of guidelines on management of the growing problem of HCC is several fold. Firstly, it aims to maximise healthcare resources when targeting large populations at risk, based on current evidence-based practice. Secondly, it allows for a standardised method of diagnosis in the era of computed tomography (CT) and magnetic resonance imaging (MRI). Lastly, it provides clinicians with a guide to the treatment of HCC.

Establishing universal guidelines for imaging diagnosis of HCC can be challenging, particularly in the lesions that do not display classical imaging features. Nevertheless, imaging diagnosis of HCC is important because it is noninvasive, given that the incidence of needle tract tumour seeding following biopsy of HCC is small but not negligible (overall 2.7%, or 0.9% per year) [5], while the risk of significant haemorrhage-related complications following image guided liver biopsy is 0.5% (based on a retrospective review of 3636 percutaneous core biopsies performed at a single institution) [6]. Furthermore, it allows for proper delineation of extent of disease, which impacts on the type of treatment, including local ablative therapy, such as radiofrequency ablation, transhepatic arterial chemo-embolisation (TACE), surgery or transplant. It can allow for accurate localisation of tumour foci, making it possible for local ablative therapies and proper surgical planning.

The purpose of this paper is to review the similarities and differences between the more recent guidelines on radiological diagnosis of HCC as proposed by the APASL and the AASLD.

## 2. Radiological Diagnosis of HCC

The use of imaging in HCC diagnosis can be best divided into two main categories. The first is in the surveillance of patients at high-risk for developing HCC. The second is in the diagnosis of HCC based on an abnormal screening test.

## 3. Surveillance

Prospective screening of patients at high-risk of developing HCC increases the proportion diagnosed with potentially curable disease. A screening strategy should focus on those patients with chronic HBV or HCV virus infection that has progressed to cirrhosis since more than 40% of these patients will develop HCC [7].

As for the time interval between surveillance tests, both the AASLD and APASL recommend measurement of serum alpha-fetoprotein (AFP) levels combined with grey-scale ultrasound (US) of the liver for surveillance of HCC [3, 4] at 6-monthly intervals for HBV carriers and patients with chronic hepatitis, since it has been shown on metaregression analysis to demonstrate a significantly higher sensitivity for early HCC with US every 6 months than with annual surveillance [8, 9]. Although detailed discussion regarding the serological markers for HCC are beyond the scope of this paper, brief mention needs to be made with regards to AFP since it is the single most commonly used serologic marker for HCC.

As with all diagnostic tests, the sensitivity profile of AFP is reduced when a higher threshold is applied in order to improve specificity. On its own, AFP is not sufficient as a screening test for HCC [10]. Taking the most commonly report cut-off of 20 ng/mL, AFP carries a sensitivity of 41–65% and a specificity of 80–94% [11]. Particularly in high-risk patients, it has a low positive predictive value of around 25% [12]. 

US screening is superior to alpha-fetoprotein assay for detection of HCC [13]. Combined AFP and US further increases detection rate [14]. As such, combined use of AFP monitoring and US is recommended, in patients with chronic HCV [15, 16] as well as HBV, where it has been found to reduce mortality (37–41%) [17, 18]. Despite the higher sensitivity and specificity of CT and MRI for detection of HCC [19], these have not been validated for and are therefore not currently recommended for screening.

## 4. Imaging Diagnosis

A feature common to the APASL and AASLD guidelines is that the recommendations for imaging diagnosis of HCC are to be interpreted in the context of patients at high-risk for HCC [3, 4]. This would include patients with liver cirrhosis and those with chronic HBV infection without definite cirrhosis. It is important to make this distinction, since the guidelines may not necessarily apply to the general population.

### 4.1. Classical Imaging Features

There is little disagreement between the consensus guidelines of the APASL and the AASLD on the definition of imaging features of classical HCC. The presence of arterial hypervascularity and washout are generally considered to be highly specific for the diagnosis of HCC, and shall henceforth be referred to as “classical imaging features” [20]. In particular, this enables differentiation from intrahepatic cholangiocarcinoma, which shows delayed enhancement [21]. At the time of the EASL guidelines in 2001, the importance of “washout” was not fully appreciated, hence not included. However, this is now specifically emphasized as a crucial feature in the APASL and AASLD guidelines. 

Arterial hypervascularity is defined as increased enhancement of the lesion in the hepatic arterial phase of imaging relative to the background liver. This is based on the fact that HCC receives predominant vascular supply via the hepatic artery. A precontrast and a dynamic postcontrast scan of the liver is necessary to demonstrate this on imaging. 

“Washout” of the lesion is based on the fact that HCC contains predominantly arterial blood and so, by the time portal venous and delayed images are acquired, the lesion is observed to be hypoattenuating on CT (or in the case of US, “hypoechoeic” and in the case of MRI, “hypointense”) to the surrounding liver at the portal venous or equilibrium phase. Washout can be explained in terms of tracer kinetic modeling of a lesion with high proportion of intravascular space [22] For demonstration of washout, the delayed phase has been shown to be superior to the portal venous phase, both for CT and MRI; this is estimated at 2-3 minutes following injection of intravenous contrast agents [23, 24]. The timing of the scans are important, and this has led to the recommendation that imaging be performed in specialised centers [25]. 

The presence of elevated AFP greater than 200 ng/mL is no longer required under the revised AASLD guidelines, as it is recognised that there are inherent false-positives (in cirrhotic patients) and false negatives [3, 25]. Detailed discussion on the role of AFP is beyond the scope of this paper, although the limitations of AFP as a serologic marker for HCC has previously been alluded to.

Despite the abundant use of multidetector row technology, CT may underestimate the extent of disease in around 50% of cases [26]. Although it has been established in that MRI is superior in the detection of HCCs, particularly the lesions smaller than 2 cm in size [27, 28], neither the APASL nor the AASLD recommends the use of MRI over CT for staging of disease. In the study by Pitton et al. where direct comparison between MRI and 64-row CT, MRI was significantly more sensitive in detecting tumour nodules [29]. However, the decision to use MRI over CT can be limited by its relatively high cost and technical demand.

### 4.2. Atypical Imaging Features—AASLD Guidelines

Most of the differences between the AASLD and APASL guidelines for the radiological diagnosis of HCC lie in the approach to lesions that do not demonstrate the classical imaging features of HCC. The AASLD essentially does not recognise use of nonvascular imaging criteria, and in the absence of the classical arterial hypervascularity and venous washout pattern of HCC, further evaluation is necessary. While this makes the AASLD guidelines more applicable to transplant guidelines (Milan and UCSF criteria), where diagnoses were based on vascular enhancement pattern of HCCs [30, 31], it may also lead to understaging of disease [3]. 

**Figure 1:**
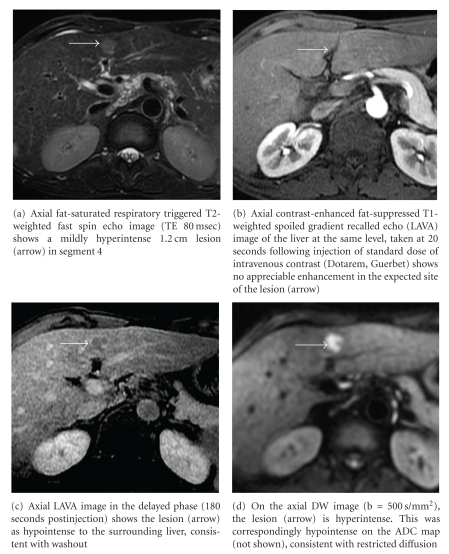
HIV positive patient with chronic HBV infection without known liver cirrhosis. By the AASLD and APASL guidelines, this lesion would require further evaluation. CT done prior to the MRI also failed to demonstrate arterial hypervascularity. Note, however, that the lesion showed suspicious features on T2-weighted and DW imaging. The lesion was biopsied percutaneously under ultrasound guidance and showed to represent a well-differentiated HCC.

Often, the lesions that do not conform to the classical imaging features are better differentiated and smaller than 2 cm in size. These “early” HCCs have been shown to contain not only fewer portal tracts but also fewer arterioles [32]. This is reflected by their atypical imaging appearances, where 87% of well-differentiated lesions and 41–62% of lesions smaller than 2 cm showed either absence of arterial hypervascularity, venous washout, or both (Figure 1) [33, 34]. Importantly, these are the lesions that should be the target of surveillance and diagnoses, since they can be ablated with high likelihood of cure [25].

Conversely, for the larger lesions, even in the absence of the classical imaging features, size alone is a risk factor [34]. In the series by Yu et al. in patients with known HBV-induced cirrhosis, lesions with a spherical contour greater than 2 cm were found to have high malignant potential, despite lack of arterial hypervascularity [35]. Indeed, the classical enhancement features for HCC in large lesions may be confounded by the presence of central necrosis and lesion heterogeneity (“nodule-in-nodule” appearance) [36]. 

In the revised AASLD guidelines, lesion size continues to predominate, though less so compared to the earlier edition. In the earlier AASLD guidelines, any lesion greater than 2 cm in size and demonstrates classical imaging features can be treated without biopsy. For lesions that were between 1 to 2 cm in size, two imaging modalities, rather than one, with classical features were needed to confirm the presence of HCC and avoid biopsy. This has been recently revised such that any lesion larger than 1 cm that demonstrate the classical pattern of HCC can be deemed as such and treated accordingly without biopsy. This is because as with the larger lesions, the approach of using a single imaging technique for lesions that are between 1 to 2 cm yields acceptable results [37–39]. 

In the presence of atypical findings from a single imaging test (CT or MRI), the AASLD recommends a different imaging modality (CT or MRI) for further assessment. This has been validated by Khalili et al. in which single imaging scans were found to have similar specificity (91–99%) to two coincidental positive scans (91–100%) with much less resource utilization and higher sensitivity (74–89% versus 53–62%) [38]. However, if atypical findings are again demonstrated, biopsy is recommended. Biopsy restores the specificity of imaging to 100% where any of the findings are atypical [40]. Note that contrast-enhanced ultrasound (CEUS) is not considered to be specific enough (besides the fact that the CEUS agents are not commercially available in the United States) and is excluded from the revised AASLD guidelines [3].

Even though the majority of cirrhotic nodules smaller than 1 cm are benign [3], Kim et al. found that in patients with mild cirrhosis related to HBV, HCCs were present in two-thirds of hypervascular lesions smaller than 1 cm [41]. As such, in lesions smaller than 1 cm, the specificity of imaging for HCC is limited [42], and based on AASLD guidelines, these cannot be regarded as HCC, regardless of the enhancement pattern. A foreseeable problem with imposing this size criteria is that it can pose dilemma in clinical practice, since it has been shown that subcentimetre lesions can be diagnosed, particularly with MRI [43]. 

 Instead of aggressively chasing the diagnosis through biopsy for lesions smaller than 1 cm (which in itself can be technically challenging due to size), close interval followup in 3 months using the modality that best depicts the lesion is recommended. Here, the guidelines may be debated. It has been suggested that for among hypervascular nodules smaller than 1 cm, those smaller than 5 mm, are subcapsular in location, wedge shaped, or ill defined (more likely to represent vascular shunts) a 6-month followup is sufficient, but when the nodule is round, oval, intraparenchymal, or in a dominant mass (more suspcious for HCC), closer imaging followup at 3-monthly intervals should be performed [44]. This may reduce unnecessary imaging but requires further validation. Typically, nodules are declared benign only if they regress or remain stable for two years, since HCC nodules can grow very slowly [2].

### 4.3. Atypical Imaging Features—APASL Guidelines

The APASL guidelines approach the atypical lesions in different manners. Essentially, these focus on Kupffer cell density as a marker of benignity. It has been shown that Kupffer cell density decreases with dedifferentiation of the cirrhotic nodule [45, 46] and is reflected by two different classes of imaging contrast agents. The first is a second generation CEUS agent containing perfluorobutane microbubbles (Sonazoid, GE Healthcare); its use is currently limited as it is not available outside of Japan. The other is superparamagnetic iron oxide (SPIO) MR contrast agents, namely ferucarbotran (Resovist, Bayer) and ferumoxide (Feridex, AMAG pharmaceuticals). Since normal liver tissue contains Kupffer cells, which are in turn part of the reticuloendothelial system, malignant lesions can be reliably differentiated from nontumourous liver based on the fact that they do not contain Kupffer cells. 

The APASL guidelines basically divides the atypical lesions into those that are hypervascular (and do not demonstrate washout) and those that are hypovascular (and do not show arterial hypervascularity). For hypervascular lesions that do not demonstrate washout, early HCCs can be reliably differentiated from focal nodular hyperplasia and arterioportal shunts based on differential uptake of Kupffer-specific contrast agents. On the parenchymal phase of imaging, HCCs should appear as unenhanced areas on CEUS and as T2*-hyperintense lesions on SPIO-enhanced MRI. However, a foreseeable limitation is in the characterisation of other hypervascular malignancies, such as neuroendocrine carcinoma metastases. 

The approach to the hypovascular lesion is a little more complex, while at the same time, the differential list for this includes a larger group of hepatic malignancies, including intrahepatic cholangiocarcinoma and metastases. Basically, if the lesion is initially shown to be hypovascular on CT and MRI, CEUS may be attempted to demonstrate enhancement in the hepatic arterial phase. If this is shown to be true, the lesion may be deemed HCC. Alternatively, if Kupffer-specific imaging demonstrates a relative lack of uptake, the lesion can be regarded as HCC. Again, the limitation of such an approach is that the other concomitant hypovascular lesions such as adenocarcinoma metastases are not definitely excluded.

Although CT arterial portography and CT hepatic arteriography (CTPA and CTHA) are considered to be significantly more sensitive for demonstrating the early vascular changes in small HCCs [47], these are invasive and the expertise for these procedures is not readily available in many centres around the world. 

The ensuing sections will briefly review various imaging modalities used in diagnosis and assessment of HCC; some of these are included in the current APASL guidelines, the rest are meant to inform the reader of recent advances in imaging of HCC that may potentially be integrated into future diagnostic imaging algorithms.

### 4.4. Kupffer Specific Imaging: Sonazoid CEUS and SPIO Agents

Given that the APASL recommends the use of Kupffer-specific agents (Sonazoid and SPIO agents) for lesion characterisation, a more detailed discussion on the utility of these contrast agents needs to be made. However, in part because neither Sonazoid nor currently commercially available SPIO agents are approved by the United States Food and Drug Administration (FDA) for clinical use, these are not included under the diagnostic algorithm by the AASLD. CEUS on its own is an accepted imaging modality for HCC diagnosis under the APASL guidelines and this has been validated even for lesions smaller than 2 cm [37]. Jang et al. showed that the sensitivity, specificity, and accuracy of CEUS for diagnosing HCC was 87%, 100%, and 93%, respectively, [48].

**Figure 2:**
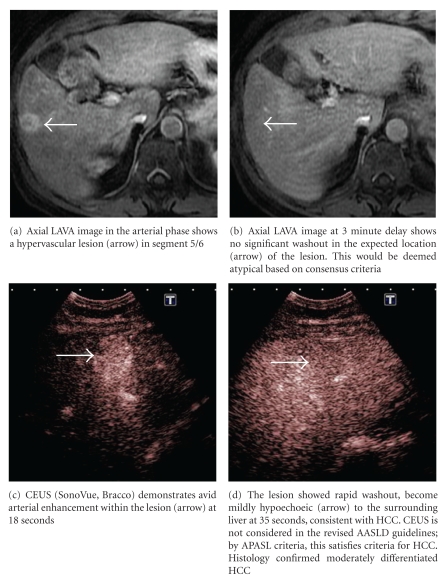
Patient with chronic HCV infection found to have a 2 cm hypoechoeic nodule on surveillence ultrasound scan. Both CEUS and multiphasic contrast-enhanced MRI were performed.

Inherently, the enhancement patterns of lesions on CEUS reflect tumour microvascular morphology, making it a valuable method for predicting the histological grade [49] while providing valuable information for antiangiogenic therapy [50]. The keys limitations of CEUS are that it is operator dependent and has decreased sensitivity in obese patients and lesions far from the skin surface [51]. Furthermore, the phenomenon of “washout” on CEUS is less specific for HCC than it is with CT or MRI, due to significant overlap between nearly all malignant and some benign lesions. Washout in CT or MRI is determined by contrast dynamics in both the intravascular space and the interstitium whereas CEUS washout is predominantly related to contrast dynamics in the intravascular space (Figure 2). Moderately differentiated HCC generally shows classic enhancement features, while well-differentiated and poorly differentiated tumours account for most atypical variations [52]. 

Adding Kupffer-specific phase imaging to CEUS protocols may yield additional information that can be used to further assess histologic grades of tumour and enable better characterisation among dysplastic nodules, moderately-differentiated and poorly differentiated HCCs [45]. As with SPIO imaging, Kupffer-specific imaging enables detection of all moderately and poorly differentiated HCCs [46]. The reader should however bear in mind that these findings are read in the context of patients at high-risk for HCC development. Kupffer phase imaging itself remains nonspecific, since even benign lesions, such as haemangiomas, that do not contain Kupffer cells, will appear as hypoechoeic on Kupffer-specific phase of CEUS.

Similarly, use of SPIO has been shown in multiple studies to improve accuracy of MRI for detection of HCCs. However, detailed discussion of the SPIO agents will be avoided since these are currently out of production, except to say that experience with SPIO agents thus far had been promising and that it potentially improves imaging detection of HCCs [40, 53, 54]. Combined gadolinium chelate and SPIO MRI, termed “double contrast” MRI, is technically more cumbersome, even though it appears to increase the tumour to liver contrast to noise ratio, and therefore sensitivity, over multiphasic CT [55, 56] routine Gd-enhanced MRI [57], or SPIO-enhanced MRI [58, 59].

### 4.5. Imaging of Tumour Thrombosis in HCC: Worth a Look? 

Although important for staging and treatment decision making, assessment of portal vein thrombosis for tumour involvement is currently not considered in both the APASL or AASLD guideline recommendations. Image guided percutaneous biopsy of suspected portal vein tumour thrombosis is feasible but invasive [60]. It may be possible to apply the same (AASLD or APASL) criteria used in diagnosis of HCC nodules to the vessel of interest to determine tumour involvement, but this does not appear to have been well studied. Separate guideline recommendations may be necessary.

Various noninvasive techniques have been investigated, and among them, CEUS appears to show fairly good success [61], superior to that of CT [62]. CEUS itself carries a sensitivity of 88% for diagnosing malignant portal vein thrombosis [63]. Combining CEUS and CT, Sorrentino and colleagues found 100% positive predictive value if both imaging modalities demonstrated arterial hypervascularity within the thrombi. In that study, the overall sensitivity of imaging for malignant thrombosis was 75% [64]. In the small series by Sun et al. 18-FDG PET may discriminate between benign and malignant portal vein thrombi but larger numbers are necessary [65]. Based on the absolute ADC values, diffusion-weighted (DW) MRI shows promise for discriminating between bland and tumour portal vein thrombi [66], but has not been fully validated.

### 4.6. Hepatocyte-Specific MRI Agents, DW MRI and Positron Emission Tomography (PET): On the Horizon? 

Functional imaging of HCC is fast becoming a reality and a brief mention of some of these techniques shall be made. Hepatocyte-specific gadolinium chelate agents are relatively new and are not currently included in the guideline recommendations. Gadoxetic acid (Gd-EOB-DTPA, Primovist, Bayer) and gadopentetate dimeglumine (Gd-BOPTA, Multihance, Bracco) are two such contrast agents that have been shown to improve diagnosis of HCC, showing diagnostic performance similar to or better than SPIO [67, 68] and comparable to double contrast MRI [69].

Hepatocyte-specific gadolinium chelate agents allow for multiphasic dynamic contrast-enhanced MR imaging to be combined with the hepatocyte-specific phase. These require delayed scanning of approximately 20 minutes in the case of Gd-EOB-DTPA and 60–120 mins in the case of Gd-BOPTA to provide maximal lesion to liver contrast [70]. Specifically, they may be used to differentiate HCCs from the arterial enhancing pseudolesions and are recommended for diagnosis of focal nodular hyperplasia [71, 72]. Like SPIO agents, they may allow for characterisation of the degree of tumour differentiation [73].

**Figure 3:**
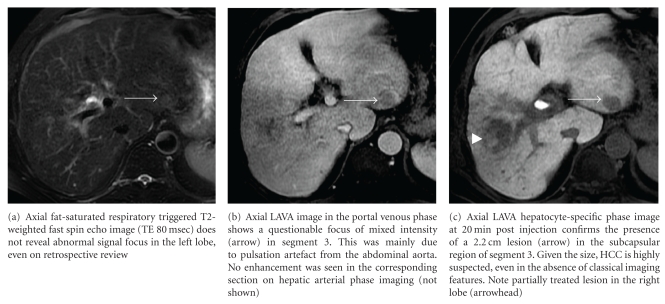
Chronic HBV patient with known multifocal HCC presumed to be confined to the right lobe, completed one session of TACE. US suggested possible nodule in the left hepatic lobe, but this was occult on multiphasic CT. MRI with standard dose of Gd-EOB-DTPA was performed.

Gd-BOPTA-enhanced MRI with hepatocyte-specific phase imaging improves diagnosis over routine multiphasic CT or MRI [74], with quoted sensitivity and specificity rates of 97% and 88%, respectively, [75, 76] (Figure 3). Gd-EOB-DTPA-enhanced MRI is also superior to CT, with reported accuracy of 0.88, compared to 0.74 in CT [77–79]. Between the two agents, Gd-EOB-DTPA was more sensitive than Gd-BOPTA for HCC detection (86% compared to 64%) [80], perhaps related to the fact that the extent of hepatobiliary uptake is considerably less with Gd-BOPTA (5% versus 50%).

Combining Gd-EOB-DTPA-enhanced MRI and Sonazoid CEUS detected 73% of the nodules not detectable by multiphasic CT [81]. It may also be combined with diffusion-weighted (DW) MRI to improve diagnosis [82]. However, assessment for lesions smaller than 1 cm can be still poor (sensitivity of 29–43%) [83], and hence further experience is necessary with these hepatocyte-specific agents before they are included in imaging guidelines. 

DW MRI studies the random motion of water molecules and shows promise for detection and characterisation as well as posttreatment assessment of tumours [84]. It improves MR detection of HCCs, particularly in lesions smaller than 2 cm [85], with sensitivities of 84–98% compared to 76–85% for multiphasic MRI alone [86–88]. Potentially, objective measurement of the apparent diffusion coefficient (ADC) may allow for distinction between the different tumour grades [89, 90]. It can be combined with SPIO-enhanced MRI, raising sensitivity from 66% to 70%, while maintaining high specificity of 98% [91]. DW MRI also shows potential for assessment of treatment response to local ablative therapies [88, 92]. Its role in the diagnostic algorithm is not certain at this point, although, given the promising results and its ease of implementation in routine clinical practice (due to fast acquisition times, no needs for additional hardware and ease of interpretation), incorporation into future guidelines is anticipated.

^18^Fluorodeoxyglucose (FDG) PET is generally accepted to have low sensitivity (50–68%) for intrahepatic HCC [93–95] and is therefore not considered to be useful for diagnosis of HCC, except perhaps in cases of poorly differentiated HCC where it may show better results [96]. Dual tracer imaging with the addition of ^11^C-acetate improves sensitivity for intrahepatic disease from 37–49% for 18-FDG and 11-C alone to 90% when combined [97]. The role of 18-FDG is limited to evaluation of extrahepatic disease [98], with sensitivity of 13–84%, depending on the size of the lesions [99]. Newer tracers such as ^18^F-choline [100] and^18^F-thymidine [101] have shown slightly better results, but further experience is needed. At present, PET plays a small role in imaging assessment of HCC, but tumour-specific tracers may be the key to its use in future.

## 5. Summary

Our understanding of the pathophysiology of HCC has improved tremendously over the past decade. This has been paralleled by advancements in US, CT and MRI technology, along with development of various Kupffer- and hepatocyte-specific imaging contrast agents. As the treatment of HCC becomes more sophisticated, a greater need for highly accurate diagnosis is necessary. The consensus recommendations by the AASLD and APASL on the radiological diagnosis of HCC underscore the push for noninvasive diagnosis of HCC in avoidance of biopsy. 

While there is general consensus with regards to the surveillance for HCCs in high-risk patients, pertinent differences in the recommendations for imaging diagnosis of HCC exist. These reflect the differences in the availability of diagnostic imaging resources in different regions. For example, Sonazoid is not available for use outside of Japan and is therefore unique to the APASL guidelines. In a way, they also point to differences in practice patterns and the controversies in our understanding of “early” HCC. The AASLD guidelines demand that the classical enhancement features of HCC are demonstrated, accepting that this may limit sensitivity; biopsy is regarded as a means to restore sensitivity. On the other hand, the APASL guidelines emphasizes the use of Kuppfer specific imaging techniques to improve diagnostic performance.

With rapid and continual improvement in diagnostic imaging modalities and validation of these guidelines, further refinements to the diagnostic algorithm can be expected in the near future. At present few of the established techniques have fallen out of favour; SPIO agents are on the decline due to decreased clinical usage, while double contrast MRI, CTHA and CTAP are cumbersome to perform and not compatible with routine clinical practice. 

Hepatocyte-specific MRI contrast agents are increasingly used in the United States, Europe and parts of Asia, as well as DW imaging, which is now already widely applied in routine clinical practice, demonstrate great promise to improve current methods of imaging diagnosis. However, before these can be incorporated into the imaging algorithms, validation of their utility is necessary. Similarly, the utility of imaging for other important aspects of HCC management, such as for noninvasive diagnosis of portal vein tumour thrombosis, may also need to be addressed in time to come.

##  Conflict of Interests 

The authors have no conflict of interests to declare.
